# Region-Based Analysis with Functional Annotation Identifies Genes Associated with Cognitive Function in South Asians from India

**DOI:** 10.3390/genes16060640

**Published:** 2025-05-27

**Authors:** Hasan Abu-Amara, Wei Zhao, Zheng Li, Yuk Yee Leung, Gerard D. Schellenberg, Li-San Wang, Priya Moorjani, Aparajit B. Dey, Sharmistha Dey, Xiang Zhou, Alden L. Gross, Jinkook Lee, Sharon L. R. Kardia, Jennifer A. Smith

**Affiliations:** 1 Department of Epidemiology, School of Public Health, University of Michigan, Ann Arbor, MI 48109, USA; hhabua@umich.edu (H.A.-A.); zhaowei@umich.edu (W.Z.); skardia@umich.edu (S.L.R.K.); 2 Survey Research Center, Institute for Social Research, University of Michigan, Ann Arbor, MI 48104, USA; 3 Department of Biostatistics, School of Public Health, University of Michigan, Ann Arbor, MI 48109, USA; zlisph@umich.edu (Z.L.); xzhousph@umich.edu (X.Z.); 4 Penn Neurodegeneration Genomics Center, Department of Pathology and Laboratory Medicine, Perelman School of Medicine, University of Pennsylvania, Philadelphia, PA 19104, USA; yyee@pennmedicine.upenn.edu (Y.Y.L.); gerardsc@pennmedicine.upenn.edu (G.D.S.); lswang@mail.med.upenn.edu (L.-S.W.); 5 Department of Molecular and Cell Biology, University of California, Berkeley, CA 94720, USA; moorjani@berkeley.edu; 6 Center for Computational Biology, University of California, Berkeley, CA 94720, USA; 7 Department of Geriatric Medicine, All India Institute of Medical Sciences, New Delhi 110029, India; abdey@hotmail.com; 8 Department of Biophysics, All India Institute of Medical Sciences, New Delhi 110029, India; sharmistha_d@hotmail.com; 9 Department of Epidemiology, Johns Hopkins Bloomberg School of Public Health, Johns Hopkins University, Baltimore, MD 21205, USA; agross14@jhu.edu; 10 Department of Economics, University of Southern California, Los Angeles, CA 90089, USA; jinkookl@usc.edu

**Keywords:** genetics, genomics, region-based analysis, cognitive function, whole genome sequencing, South Asian

## Abstract

**Background/Objectives:** The prevalence of dementia among South Asians across India is high among those who are 65 years and older, yet little is known about genetic risk factors for dementia in this population. **Methods:** Using whole-genome sequence data from 2680 participants from the Diagnostic Assessment of Dementia for the Longitudinal Aging Study of India (LASI-DAD), we performed a gene-based analysis on the missense/loss-of-function (LoF) and brain-specific promoter/enhancer variants of 84 genes, previously associated with AD in European Ancestry (EA). These analyses were performed separately, both with and without incorporating additional annotation weights (e.g., deleteriousness, conservation scores), using the variant-Set Test for Association using Annotation infoRmation (STAAR). We investigated associations with the Hindi Mental State Examination (HMSE) score and factor scores for general cognitive function and five cognitive domains. **Results:** In the missense/LoF analysis, without annotation weights and controlling for age, sex, state/territory, and genetic ancestry, three genes were associated with at least one measure of cognitive function (FDR q < 0.1). *APOE* was associated with four measures of cognitive function, *PICALM* was associated with HMSE score, and *TSPOAP1* was associated with executive function. The most strongly associated variants in each gene were rs429358 (*APOE* ε4), rs779406084 (*PICALM*), and rs9913145 (*TSPOAP1*). Rs779406084 is a rare missense mutation that is enriched in LASI-DAD compared to EA (minor allele frequency = 0.075% vs. 0.0015%). **Conclusions:** Missense/LoF variants in some genes previously associated with AD in EA are associated with measures of cognitive function in South Asians from India. Analyzing genome sequence data allows the identification of potential novel causal variants enriched in South Asians.

## 1. Introduction

Dementia is a group of neurological disorders characterized by cognitive impairment. In 2019, the estimated global economic cost of dementia was about $1.3 trillion USD [1]. The public health burden for dementia is borne disproportionately by lower- and middle-income countries, which harbor approximately 61% of affected individuals [1]. Over 50 million people worldwide have Alzheimer’s Disease (AD), the most prevalent form of dementia [2], and this number is projected to reach over 150 million by 2050 [3]. Cognitive decline, even without dementia, increases the need for costly personal and medical care.

While extensive research has focused on risk factors for later-life cognitive decline and dementia, there are still remaining questions regarding its etiology. For example, AD is a result of the accumulation of amyloid β plaques and neurofibrillary tangles in the brain [4]. Amyloid β and tau protein metabolism may be influenced by genetic variants that alter chemical properties or abundance of relevant proteins [5]. Heritability estimates for AD are high (60–80%) [6], indicating that the identification of AD-associated variants is critical for a deeper etiological understanding. Heritability of cognitive function is also relatively high across the life course (40–80%) [7]. However, the vast majority of genetic loci for measures of cognitive function and dementia were identified from studies conducted in European Ancestry (EA) participants. A deeper exploration of the genetic factors underlying late-life cognition and dementia in non-EA populations is now needed to both identify population-specific risk variants across the genome and gauge the relative importance of previously identified loci.

With over 1.4 billion people, India is the second most populous country in the world, and the public health burden of dementia is dramatically increasing as the population both grows and ages. The prevalence of dementia among South Asians living in India varies by geographic location and sociodemographic characteristics (e.g., rural vs. urban), and is approximately 7.4% among individuals who are 60 years and older [8]. While studies have indicated that older age, lower education, diabetes, obesity, and other factors increase risk of dementia in India [9], there has been little research on genetic risk factors. Therefore, it is unclear whether the same genes that have been associated with dementia and cognitive decline in EA have a similar influence on dementia risk in South Asians. Likewise, there may be causal risk variants in known AD genes, or in other genes, that are unique to India.

Detection of rare variants associated with measures of cognitive function in non-EA populations may be difficult due to the combination of increased genetic diversity and smaller sample sizes available for genetic research, both of which lead to a loss of statistical power. Statistical power can be increased by grouping together variants within a gene or genomic region that have the same functional annotation, such as those that alter protein structure (e.g., loss-of-function (LoF) or missense variants) or those in regulatory elements (e.g., gene promoter or enhancer regions), which helps increase the likelihood of selecting probable causal variants [10].

In this study, we examined whether 84 genes previously associated with AD in EA are also associated with seven measures of cognitive function in 2680 participants from the Diagnostic Assessment of Dementia for Longitudinal Aging Study of India (LASI-DAD), a nationally representative study that includes diverse ethno-linguistic and geographic groups. From whole-genome sequence (WGS) data, we selected missense/loss-of-function (LoF) single-nucleotide variants (SNVs) and brain-specific promoter and enhancer SNVs within each gene. This work will help elucidate genetic variants associated with cognitive function in South Asians across India, which may play an important role in risk stratification, and help guide intervention and treatment plans for those at risk of dementia in India.

## 2. Materials and Methods

### 2.1. Study Population

LASI [11] is a nationally representative cohort of Indian adults who are at least 45 years of age. LASI-DAD, an ancillary study investigating risk factors for dementia, enrolled 4096 LASI participants from 18 states and union territories across India. Participants were selected using two-stage stratified random sampling across states/territories in India, with respect to cognitive impairment risk. The sampling strategy is described elsewhere [12,13]. Briefly, participants were classified as low risk or high risk for cognitive impairment based on their performance on core cognitive tests conducted in the larger LASI cohort, or on proxy reports if the participant did not complete those tests. Then, an approximately equal number of respondents in the high-risk and low-risk strata were randomly drawn from each state/territory with a target sample size proportionate to the population size. Participants underwent neurocognitive testing with tests logically and culturally adapted from tests present in the Harmonized Cognitive Assessment Protocol (HCAP) [12], informational interviews, and a blood draw to extract DNA for whole genome sequencing. A total of 2680 participants with complete genotype and cognition data were included in the analysis.

### 2.2. Whole-Genome Sequence Data

Whole genome sequencing (WGS) at an average read depth of 30× was performed by MedGenome, Inc. (Bangalore, India) using DNA extracted from blood samples from 2762 LASI-DAD participants. Genotype calling and quality control (QC) were performed at the Genome Center for Alzheimer’s Disease (GCAD) at the University of Pennsylvania [14]. Briefly, sample-level quality control included checks for low coverage, sample contamination, sex discrepancies, concordance with previous genotype data, and duplicates [14]. After excluding control samples and samples with low quality and/or unresolved identity, a total of 2680 samples were retained in the analysis. At the genotype level, each genotype was evaluated and set to missing if read depth was less than 10 (DP < 10) or genotype quality score was less than 20 (GQ < 20). At the variant level, a variant was excluded if it was monomorphic, was above the 99.8% Variant Quality Score Recalibration (VQSR) tranche (the quality score was beyond the range that contains 99.8% of true variants), had a call rate ≤ 80%, or had an average mean depth > 500 reads. We further removed variants that were in low complexity regions identified with the mdust program [15]. After quality control and filtering, we retained a total of 71,109,961 autosomal bi-allelic variants that include 66,204,161 single nucleotide polymorphisms (SNPs) and 4,905,800 indels.

### 2.3. Principal Component Analysis and Genetic Relationship Matrix

We estimated genetic principal components (PCs) and the genetic relationship matrix (GRM) in GENESIS (version 2.26.0) [16,17]. For this analysis, we included variants with minor allele frequency (MAF) ≥ 5% and pruned for LD (r^2^ = 0.1, window size = 500 kb) to select independent variants. Kinship coefficients were first estimated using “snpgdsIBDKING” function. Subsequently, genetic principal components (PCs) were calculated using “PCair”, which estimates population structure while accounting for cryptic relatedness in the samples. Specifically, PCs were first estimated in a set of unrelated individuals (kinship cutoff = 0.044) to obtain robust variant weights, which were then used to project PCs in the rest of the sample. Following this, the genetic relationship matrix (GRM) was estimated using “PCrelate” by simultaneously adjusting the top 2 PCs to avoid potential confounding from population structure.

### 2.4. Measures of Cognitive Function

We analyzed seven measures of cognitive function including five cognitive domains (memory, orientation, language/fluency, executive function, and visuospatial function), general cognitive function constructed from the five cognitive domain scores, and the Hindi Mental State Exam (HMSE) score. The HMSE is a version of the Mini Mental State Exam dementia screener translated into Hindi. It is designed to be administered to participants from a population where a significant proportion of individuals are illiterate and is scored as the sum of 22 items which totals to an integer between 0 and 30, with a higher score indicating more cognitive intactness [18]. The five cognitive domain scores are factor scores of a collection of tests assigned to a broad domain of cognition as informed by the Cattell–Horn–Carroll (CHC) theory of human cognitive abilities, with composite weights and tests described elsewhere [19]. The cognitive domain and general cognitive function scores were each estimated using the item-response theory (IRT) and were normalized to a Gaussian distribution with mean of zero and variance of one in the full LASI-DAD subcohort [19].

### 2.5. Demographics and Lifestyle Factors

Sex was self-reported. Age was recorded at the time of interview. Location was defined as the participant’s report on whether they live in a rural or urban area. Literacy status was self-reported. The highest level of education the participant completed is categorized as less than lower secondary education, upper secondary education or vocational training, and tertiary education. Alcohol use was defined as having consumed alcohol within the 3 months prior to interview versus not. Smoking was categorized as having never smoked (reported never smoking), being a former smoker (reported had smoked before, but not within the 3 months prior to interview), or being a current smoker (participant had smoked within the 3 months prior to interview). Physical activity was defined as conducting vigorous physical activity every day compared to less frequently than every day. AD/dementia and psychiatric medication use were self-reported according to whether the participant currently takes the medication. Body mass index (BMI) was categorized according to the World Health Organization’s recommended thresholds for South Asians: underweight (<18 kg/m^2^), normal weight (18 to < 23 kg/m^2^), overweight (23 to <25 kg/m^2^), and obese (≥25 kg/m^2^).

### 2.6. Gene Selection

We selected a total of 84 genes from the two largest genome-wide association studies (GWAS) for AD in EA (Table S1, Additional File S1) [20,21,22,23,24,25,26,27,28], as well as *TOMM40* and *APOC1* which are proximal to *APOE* and are known to be associated with Alzheimer’s disease [22,23]. Briefly, Bellenguez et al. [20] performed a two-stage GWAS of 10 case–control studies across Europe. Stage 1 included 39,106 clinically diagnosed AD cases, 46,828 proxy AD and dementia-related disorder (ADD) cases, and 401,577 controls. Stage 2 included 25,392 AD cases and 276,086 controls. Bellenguez et al. identified 75 risk loci, of which 42 were novel. The authors then conducted pathway analysis and designed a gene prioritization algorithm to stratify loci according to their likelihood of having a causal effect on ADD risk. For this study, we selected a total of 73 genes from Bellenguez et al. [20], including those that were labeled as known loci or those that were classified as having the highest likelihood of a causal effect on ADD (tier 1). Wightman et al. [21] conducted a meta-analysis of 13 studies of EA participants across the United States and Europe. The total sample size was 1,126,563 individuals, which included 90,338 AD cases (46,613 were proxy cases) and 1,036,225 controls (318,246 were proxy controls). For this study, we selected 45 genes from independent 38 loci that reached genome-wide significance (*p* < 5 × 10^−8^). In Wightman et al., there was an overlap of 36 genes between the two AD GWAS. See Supplemental Methods for additional details on selecting genes within the identified AD loci (Methods S1, Additional File S1).

Gene boundaries were defined by GRCh38.p14 in NCBI Gene, which uses NCBI RefSeq to annotate gene positions. We selected all SNVs within the gene start and stop positions for the missense/LoF analysis. For the brain-specific promoter/enhancer analysis, we selected SNVs within a ±20 kb buffer of the gene’s transcription start site. Only genes with at least two missense/LoF or promoter/enhancer SNVs (defined below) and a cumulative minor allele count (cMAC) ≥ 5 within the region were included in the final analysis.

### 2.7. Definition of Missense/LoF and Promoter/Enhancer SNVs

We followed definitions of missense/LoF SNVs, promoter SNVs, and enhancer SNVs similar to those used by Li et al. [29]. Briefly, we used the Variant Effect Predictor (VEP) v105 [30] and LOFTEE [31] with GENCODE as the transcript annotation reference to identify missense and LoF SNVs, respectively. We additionally classified missense and LoF variants based on the confidence of their predicted function. LoF SNVs were annotated as either high confidence or low confidence using LOFTEE v1.0.3, and missense SNVs were assigned a REVEL score. REVEL scores are generated through an ensemble method to measure the pathogenicity of a missense SNV [32], with higher scores indicating greater likelihood of causing diseases. Missense SNVs with REVEL score > 0.5 are considered to have high confidence. Next, we used the WGS Annotator (WGSA) v0.95 pipeline [33] to define promoter SNVs as those that fell within ±5 kb of a gene’s transcription start site with at least one H3K4me3 annotation for brain tissues (E067-E074, E081, E082) from the ENCODE database. We defined enhancer SNVs as those that fell within ±20 kb of the gene’s transcription start site and overlapped with an enhancer defined by EnhancerFinder in the brain.

### 2.8. Annotation Selection

For both the missense/LoF and promoter/enhancer analyses, we included all SNVs that had no missing annotation weights, regardless of minor allele frequency (Methods S2, Additional File S1) [34,35,36,37,38]. Annotation weights were retrieved using WGSA v0.95 [33]. We selected a subset of annotations similar to Li et al. [29], including those that predicted deleteriousness, predicted impact on the protein, and that summarized evolutionary conservation. For missense/LoF SNVs, we used CADD_raw_rankscore, a measure of variant deleteriousness combining multiple genomic features of each variant [34]; GERP_RS_rankscore, a measure of variant conservation [35]; Eigen.phred, a measure of variant deleteriousness using an unsupervised learning method [36]; and fathmm.MKL_coding_rankscore, a score from a machine learning method incorporating other annotations to predict deleteriousness of the variant from coding variants [37]. For promoter/enhancer SNVs, we used CADD_raw_rankscore [34], GERP_RS_rankscore [35], Eigen.PC.phred [36], fathmm.MKL_non.coding_rankscore [37], and GenoCanyon_rankscore, a measure of variant conservation [38]. Genome-wide ranks of the associated annotation scores were used to generate the Phred scores (i.e., the logarithmically transformed annotation score percentiles) required for STAAR analysis.

### 2.9. Statistical Methods

All analyses were conducted in R (ver. 4.2.0). WGS data were converted from VCF files to SeqArray GDS format using SNPRelate v1.42.0 [39] and SeqArray v1.48.0 [40] R packages. We then used the variant-Set Test for Association using Annotation infoRmation (STAAR) v0.9.6.1 to perform gene-based analysis using functional annotations for missense/LoF (including all variants except low-confidence LoF), missense/LoF (high-confidence variants only), and promoter/enhancer regions separately [41]. In STAAR, linear mixed models were used to test each gene region for association with each of the seven measures of cognitive function separately, both with and without annotation weights. Model 1 was adjusted for age, sex, state or union territory, and the first ten principal components of global ancestry. Model 2 was additionally adjusted for educational attainment, rural or urban residence, and literacy status (yes/no). Each model incorporated a genetic relatedness matrix to account for relatedness between subjects, and geographic state or union territory was used to define heterogeneous variances within the linear mixed model.

For each gene, both with and without including annotation weights for the SNVs, we examined the STAAR *p*-value which is calculated from modified SKAT-(1,1), SKAT-(1,25), Burden-(1,1), Burden-(1,25), ACAT-V(1,1) and ACAT-V(1,25) tests. For analyses with and without annotation weights, separately, a Benjamini–Hochberg FDR q < 0.1 was used to declare significance. For completeness, we also report genes that reached at least a nominal significance level (*p* < 0.05). As a sensitivity analysis to investigate whether adjustment for factors related to lifestyle and medication use influenced our findings, we examined whether genes that were nominally associated with at least one cognitive measure in Model 2 were still associated after further adjustment for BMI, alcohol use, smoking, physical activity, and use of psychiatric and dementia medications.

For each gene region that was associated with a measure of cognitive function at FDR q < 0.1, we next performed a single variant analysis to identify the variants most strongly associated using a score test in STAAR. The same models from the gene-based analysis were used for single variant analysis. For the SNV with the lowest *p*-value within each identified gene, we compared the allele frequency in LASI-DAD to that found in EA samples registered in gnomAD v3 [42] to examine whether risk alleles were enriched in LASI-DAD. Regional plots of the association results were made in locuszoomr v0.3.8 and LD information was calculated on the LASI-DAD analytic sample using the unphased r^2^ from PLINK 1.9. Gene tracks for the regional plots were taken from Ensembl 113, which was retrieved through the AnnotationHub v3.16.0.

## 3. Results

The LASI-DAD analytic sample had a mean age of 69.6 (SD = 7.3) years (Table 1). The majority of participants could not read or write (56.4%), lived in rural areas (63.3%), had less than lower secondary education (75%), had normal weight (40.5%), did not consume alcohol within the 3 months prior to interview (92.6%), never smoked (76.9%), vigorously exercised less frequently than every day (83.2%), never took psychiatric medication (99.4%), and never took AD/dementia medication (99.3%). Mean HMSE score was 22.7 (SD = 5.4) (Table S2, Additional File S1).

We next characterized the distribution of the annotation weights across missense/LoF SNVs and promoter/enhancer SNVs. The missense/LoF SNVs exhibited relatively little variation for almost all annotations and tended to be high. For these annotations, which were on a scale from 0 to 1, the median score ranged from 0.97 to 0.99 (Table S3, Additional File S1). The promoter/enhancer SNVs showed greater variation across annotation weights and tended to be lower (Table S4, Additional File S1). The Eigen–Phred and Eigen–PC–Phred rank scores had more variation and relatively low weights for missense variants, but low variation and higher weights for promoter/enhancer variants, due to their calculation with different training data for each functional class of variant (Methods S2, Additional File S1).

### 3.1. Missense/Loss-of-Function (LoF) Analysis

Of the 84 genes selected for analysis, 77 had at least two missense/LoF SNVs with complete annotations and a cMAC ≥ 5, and the median number of missense/LoF SNVs across the genes was 23 (Table 2). In Model 1, 16 genes were nominally associated with at least one measure of cognitive function (*p* < 0.05, Table S5, Figure S1, Additional File S1), with 3 genes associated at FDR q < 0.1 in the analysis without annotation weights (Table 3). Specifically, *APOE* was associated with HMSE score (FDR q = 0.07), general cognitive function (FDR q = 0.04), executive function (FDR q = 0.07), and orientation (FDR q = 0.07). *PICALM* was associated with HMSE score (FDR q = 0.08), and *TSPOAP1* was associated with executive function (FDR q = 0.07). In Model 2, which additionally adjusts for rural/urban location, literacy, and education, 19 genes were nominally associated with at least one measure of cognitive function, representing 32 gene-cognitive function nominal associations (*p* < 0.05, Table S6, Additional File S1), and *PICALM* was significantly associated with HMSE score after correction for multiple testing (FDR q = 0.096) in the analysis without annotation weights (Table 3). After further adjustment for BMI, lifestyle factors, and psychiatric and dementia medication use, 17 out of 32 gene-cognitive function associations remained nominally associated without annotation weights. The FDR-significant finding from Model 2, the association between *PICALM* and HMSE score, became more significant (p_annotation_weights_ = 2.8 × 10^−5^, p_no_annotation_weights_ = 2.9 × 10^−5^).

As shown in Table 3, the results were similar when we used annotation weights. At FDR q < 0.1, *APOE* was associated with HMSE score (FDR q = 0.08) and general cognitive function (FDR q = 0.06) and orientation (FDR q = 0.0996), and *PICALM* was associated with HMSE score (FDR q = 0.08). In Model 2, at FDR q < 0.1, *PICALM* was associated with HMSE score (FDR q = 0.09).

**Figure 1:**
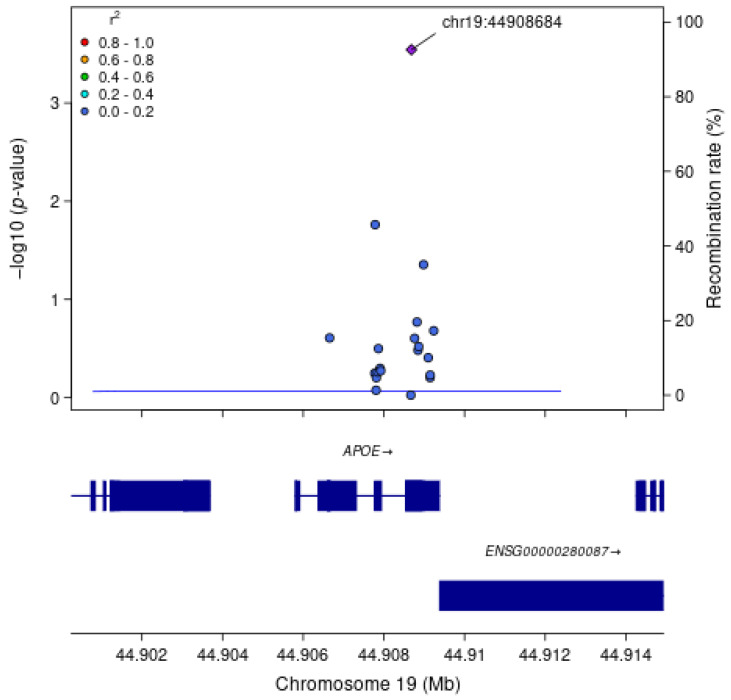
**Plot of missense/loss-of-function SNVs in *APOE* gene in Model 1 for HMSE score.** Left Y-axis: −log10 (*p*-value) from association between SNV and HMSE score, adjusting for age, sex, state/territory, the first 10 principal components of genetic ancestry, and accounting for relatedness (random effect) and heteroscedastic variances among state/territory; Right Y-axis: SNV recombination rate based on average over all reference panels from 1000 Genomes GRCh38 lifted over from hg19 from University of California, Santa Cruz; X-axis: chromosomal location and gene regions; LD information is the unphased r^2^ calculated on the LASI-DAD analytic sample; LD r^2^ color code: degree of linkage disequilibrium with index (most strongly associated) SNV, rs429358 (purple diamond). Gray points indicate no LD information present in the reference panel. No annotation weights were used to generate *p*-value.

**Figure 2:**
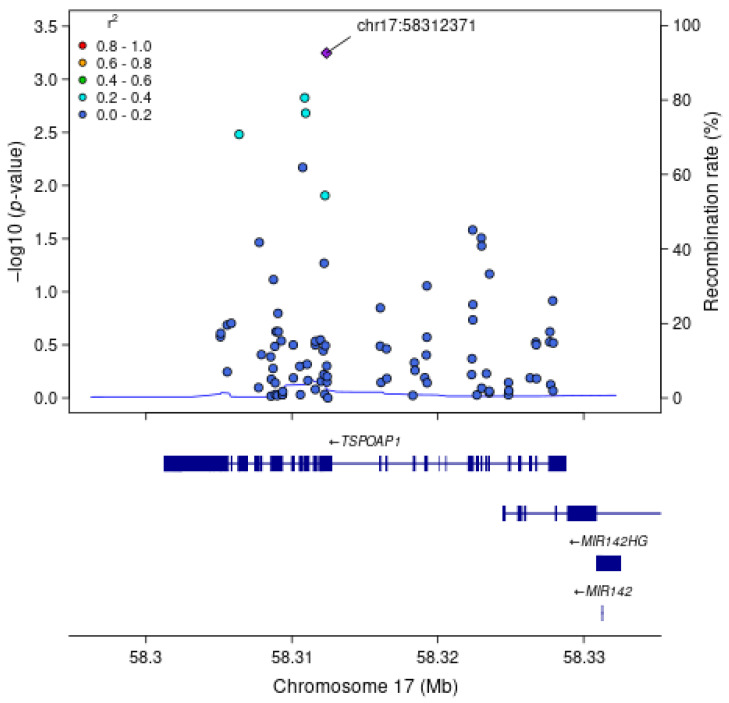
**Plot of missense/loss-of-function SNVs in *TSPOAP1* gene in Model 1 for executive function.** Left Y-axis: −log10 (*p*-value) from association between SNV and executive function, adjusting for age, sex, state/territory, the first 10 principal components of genetic ancestry; and accounting for relatedness and heteroscedastic variances among state/territory; Right Y-axis: SNV recombination rate based on average over all reference panels from 1000 Genomes GRCh38 lifted over from hg19 from University of California, Santa Cruz; X-axis: chromosomal location and gene regions; LD information is the unphased r^2^ calculated on the LASI-DAD analytic sample; LD r^2^ color code: degree of linkage disequilibrium with index (most strongly associated) SNV, rs9913145 (purple diamond). Gray points indicate no LD information present in the reference panel. No annotation weights were used to generate *p*-value.

**Figure 3:**
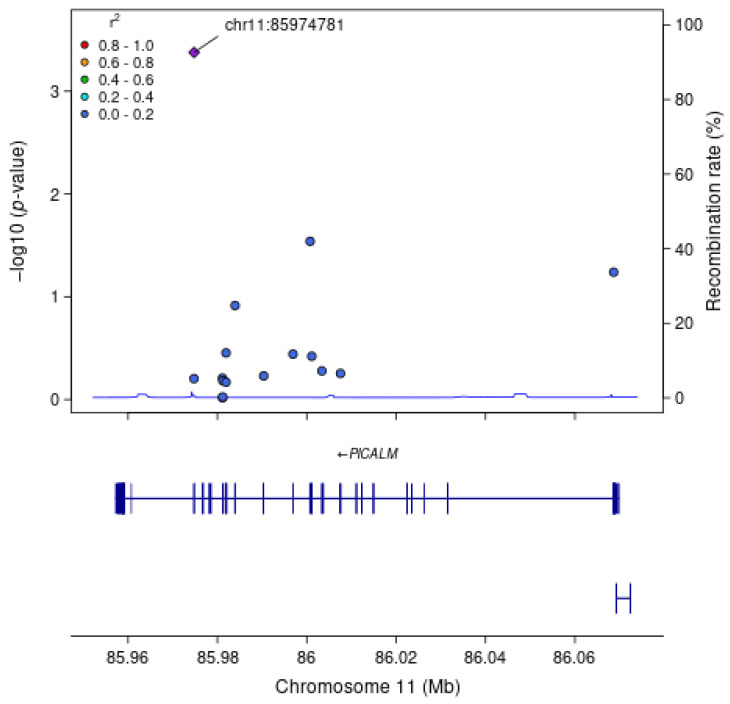
**Plot of missense/loss-of-function SNVs in *PICALM* gene in Model 1 for HMSE score.** Left Y-axis: −log10 (*p*-value) from association between SNV and Hindi Mental State Exam (HMSE) score, adjusting for age, sex, state/territory, the first 10 principal components of genetic ancestry; and accounting for relatedness and heteroscedastic variances among state/territory; Right Y-axis: SNV recombination rate based on average over all reference panels from 1000 Genomes GRCh38 lifted over from hg19 from University of California, Santa Cruz; X-axis: chromosomal location and gene regions; LD information is the unphased r^2^ calculated on the LASI-DAD analytic sample; LD r^2^ color code: degree of linkage disequilibrium with index (most strongly associated) SNV, rs779406084 (purple diamond). Gray points indicate no LD information present in the reference panel. No annotation weights were used to generate *p*-values.

For each gene associated with a cognitive measure at FDR q < 0.1, we examined associations between each SNV within the gene region, without annotation weights, and the cognitive outcome of interest (Table 4). As expected, the most strongly associated variant in Model 1 within *APOE* for all measures of cognitive function was rs429358 (HMSE *p* = 2.9 × 10^−4^, general cognitive function *p* = 1.4 × 10^−4^, executive function *p* = 4.1 × 10^−4^, orientation *p* = 2.4 × 10^−4^; Figure 1), which is the missense variant in exon 4 of *APOE* that changes cysteine to arginine and differentiates the *APOE* ε4 allele from ε2 and ε3. Removal of this SNV results in *APOE* losing significance. For *TSPOAP1* in Model 1, the most strongly associated variant with executive function was rs9913145 (Model 1 *p* = 5.7 × 10^−4^), a missense variant in exon 17 that changes glutamine to arginine (Figure 2). This variant had an MAF of 0.15 in LASI-DAD, and an MAF of 0.12 in EA samples in gnomAD, which indicates that the minor allele is relatively common both in LASI-DAD and in EA populations. In *PICALM* in Model 1 and Model 2, the most strongly associated SNV with HMSE was rs779406084 (Model 1 *p* = 4.2 × 10^−4^, Model 2 *p* = 1.6 × 10^−4^), a missense variant in exon 19 that changes threonine to methionine (Figure 3). Rs779406084 was in high LD with all missense/LoF SNVs in *PICALM* (|D’| = 1) but was not correlated with any other missense/LoF SNV in the gene (r^2^ << 0.2) including with the one other missense/LoF SNV with *p* < 0.05. This SNV has a CADD score of 24, indicating that it is in the top 0.4th percentile of all deleterious SNVs. It also has a MAF of 7.5 × 10^−4^ in LASI-DAD. While very rare, this variant occurs more often in LASI-DAD compared to EA samples in gnomAD (EA MAF = 1.5 × 10^−5^).

### 3.2. High-Confidence Missense/LoF Analysis

Of the 84 genes analyzed, 35 had at least two high-confidence LoF or missense SNVs with REVEL > 0.5 and cMAC ≥ 5. In Model 1, six genes were nominally associated with at least one measure of cognitive function (*p* < 0.05, Table S7, Figure S2, Additional File S1), but none were significant after correction for multiple testing (FDR q > 0.1). In Model 2, six genes were also nominally associated with at least one measure of cognitive function, representing six gene-cognitive function nominal associations (*p* < 0.05, Table S8) with three genes overlapping from Model 1 (*ABI3*, *APOE*, and *INPP5D*), but none were significant after correction for multiple testing (FDR q > 0.1). After further adjustment for BMI, lifestyle factors, and psychiatric and dementia medication use, two out of six gene-cognitive function associations remained nominally associated without annotation weights (Table S8). For both analyses, the results did not change substantively when annotation weights were incorporated.

### 3.3. Promoter/Enhancer Analysis

Of the 84 genes analyzed, 77 had at least two brain-specific promoter or enhancer SNVs with complete annotation weights and cMAC ≥ 5, and the median number of brain-specific promoter/enhancer SNVs across the genes was 93 (Table 2). In Models 1 and 2, 21 and 22 genes, respectively, were nominally associated with at least one measure of cognitive function without annotation weights (*p* < 0.05), but none were associated after multiple testing correction (all FDR q > 0.1, Tables S9 and S10, Figure S3, Additional File S1). There was a total of 32 gene-cognitive function nominal associations in Model 2. After further adjustment for BMI, lifestyle factors, and psychiatric and dementia medication use, 18 out of 32 gene-cognitive function associations remained nominally associated without annotation weights (Table S10). When we incorporated annotation weights, 18 genes in Model 1 and 22 genes in Model 2 were nominally associated with at least one measure of cognitive function, but again none were associated after multiple testing correction (all FDR q > 0.1, Tables S9 and S10, Additional File S1). In Model 1, *USP6NL*, *INPP5D*, and *KAT8* were no longer nominally associated with any measure of cognitive function after the incorporation of annotation weights. In Model 2, *EGFR* and *APOE* were no longer nominally associated with any measure of cognitive function after incorporation of annotation weights, but *APOC1* and *SORT1* became nominally associated.

## 4. Discussion

We performed a gene-based analysis examining the association between missense/LoF and brain-specific enhancer and promoter variants in previously identified AD-associated genes and seven measures of cognitive function in South Asians across India. Using only missense/LoF variants, three genes were associated with at least one measure of cognitive function after multiple testing correction, including *APOE* with multiple cognitive measures. However, no genes were associated with the brain-specific promoter and enhancer analysis. The most strongly associated variants were missense SNVs with high-predicted deleteriousness. One of the most significantly associated missense variants was very rare; yet it appeared to be enriched in LASI-DAD compared to EA samples in public databases.

We found that *APOE* is significantly associated with HMSE score, general cognitive function, executive function, and orientation in the missense/LoF analysis before adjusting for sociodemographic factors. Apolipoprotein E (APOE) facilitates cholesterol and phospholipid transfer between cells, and complexes with amyloid β proteins in the brain for removal, inhibiting the amyloid β plaque formation necessary for AD onset [43]. *APOE* alleles confer different risks for Alzheimer’s disease. Relative to the ε3 allele, ε4 is associated with increased risk of Alzheimer’s disease in EA [44], and the ε4/ε4 genotype is also associated with cognitive decline in those with Alzheimer’s disease [45]. In our analytic sample from LASI-DAD, the ε4 allele frequency is estimated to be approximately 10.9%, which is less than the reported frequency among EA samples in the US (14%) [46], and while not common, is still frequent. The ε4 allele has somewhat different associations with AD risk across races/ethnicities, with ε4 and associated variant effects being stronger in EA populations compared to African Americans [47]. Rs429358 is used to differentiate between ε3 and ε4 alleles in *APOE*, and has a CADD score of 16.6, which places it in the top 2nd percentile of deleterious SNVs. Although previous studies with a subset of the current LASI-DAD sample did not find an association between cognitive function and rs429358 [17,48], this was likely, due to smaller sample size and/or less regional variation in the previous studies. Our reported associations with *APOE* are not surprising, as working memory and executive function deficits are often early markers of AD [49], and other cognitive domains, such as memory and visuospatial function, are often affected later in the development of AD. In light of this, associations between *APOE* and cognitive domains outside of memory and executive function may not have been present in this younger sample.

Phosphatidylinositol binding clathrin assembly protein (PICALM) facilitates endocytosis of APP [50], which is needed to form β-amyloid plaques that lead to AD. Given its direct involvement in β-amyloid transport, it is reasonable that *PICALM* is associated with HMSE score, a dementia screener, which likely identifies cases of AD and other dementia that have progressed. However, it is not clear why *PICALM* showed no associations with specific cognitive functions typically affected prior to dementia onset. *PICALM* was found to be associated with cognitive function in EA samples [51]. *PICALM* variants identified in EA have had mixed associations in East Asian samples, with which the South Asian population of India shares ancestry [52]. For example, some variants identified as associated with AD in a large meta-analysis of Chinese GWAS near *PICALM* [53] were not found to be associated in smaller Indian studies [54]. We found that the sentinel SNV in *PICALM* for HMSE score is rs779406084, a very rare variant with a high CADD score at 24, placing it in the top 0.2th percentile of all deleterious SNVs. To our knowledge, this is the first study that reports an association with rs779406084 and cognitive function. This is likely due to the rarity of this variant in EA samples (MAF = 1.5 × 10^−5^), and an association was likely found in our study due to its comparatively higher frequency in LASI-DAD (MAF = 7.5 × 10^−4^). Further work is needed to elucidate the specific effects of this variant on the protein and replication in other cohorts is needed.

Translocator protein (TSPO) associated protein 1 (TSPOAP1) regulates calcium channels in nerve synapses [55]. It interacts with the protein TSPO which is involved in inflammation pathways [56]. Inflammatory processes were previously associated with changes in executive function in cardiac patients [57], which may support the association between *TSPOAP1* and executive function, but not other cognitive domains, in our study. *TSPOAP1* variants were associated with AD in a large transethnic AD GWAS [58]. The sentinel SNV of *TSPOAP1* in LASI-DAD was rs9913145, which has a CADD score of 1.12, indicating that it is not strongly deleterious. This variant is slightly more common in LASI-DAD (MAF = 0.15) compared to EA samples (MAF = 0.12). To our knowledge, this variant has not otherwise been reported to have an association with cognitive function or dementia. Given the relatively low CADD score of the variant and the relatively common frequency in EA samples, this variant may tag a haplotype specific to South Asians within *TSPOAP1* that is associated with executive function.

We found no associations between brain-specific promoter and enhancer SNVs within the known AD genes and any of the measures of cognitive function in our sample after multiple testing corrections. This is likely due to promoter/enhancer SNVs having more subtle effects on AD gene expression compared to the potentially more deleterious effects from missense/LoF SNVs. We also found that annotation weights did not substantively change our analysis results. This may be because the missense/LoF variants had relatively small variance in their annotation weights and tended to be high, resulting in little additional statistical information.

We found many genes that were nominally associated with each measure of cognitive function in the missense/LoF analysis and brain-specific promoter/enhancer analysis. Genes associated with multiple cognitive measures include *ADAM17*, *OTULIN*, and *ABCA7*, which are involved in amyloid-β metabolism or in immune signaling [43,59,60]. In both Model 1 and Model 2, *ABCA7*, *ADAM17*, *APOE*, *OTULIN*, and *TSPOAP1* were all at least nominally associated with three or more measures of cognitive function. These genes all play a role in cholesterol and APP metabolism (*ABCA7*, *ADAM17*, *APOE*) or are involved in inflammation pathways (*OTULIN*, *TSPOAP1*). *ABI3* and *APOE*, both nominally associated with at least one cognitive function in the high-confidence missense/LoF analysis, were previously noted to have deleterious effects on dendrites through β-amyloid metabolism [61,62,63,64]. *ABI3* was nominally associated with visuospatial cognitive domain across both models, which may be supported by findings that AD may affect visuospatial processing through β-amyloid plaques [65]. Further, several genes nominally associated with at least one cognitive function in the promoter/enhancer analysis were related to immune system signaling (*APOE* [66], *PLGC2* [67], *SCIMP* [68], *BLNK* [69]). However, these genes had no consistent associations with the cognitive domains which may reflect more diverse downstream consequences associated with immune responses that operate through signaling molecules. In general, we observed more nominal and FDR-significant associations before adjusting for sociodemographic factors. It is possible that the sociodemographic variables, which are patterned by geography, are correlated with allele frequencies in the genes examined.

Similar gene-based analysis studies were conducted in EA samples. One large genome-wide gene-based AD study conducted in the UK BioBank on different categories of rare missense/LoF variants found that three gene regions were associated with AD parent proxy cases, including *TOMM40*/*APOE* [70]. Notably, the detection of these regions depended on resolving variants into categories of high confidence and predicted loss-of-function effects. Another gene-based AD analysis conducted in the ADES-FR study found that protein-truncating rare variants and strictly damaging rare variants in *TREM2*, *ABCA7*, and *SORL1* were associated with early-onset AD, but not with late onset AD [71]. Given that the previous studies focused on very specific classes of rare variants in EA samples, it is no surprise that these genes were also at least nominally associated in our study.

Although many genes were nominally associated in our analysis, few genes were significant after correction for multiple testing. This could be in part because we selected genes associated with AD, which may have weaker effects on cognitive function changes that precede AD. Further, the genes were identified through GWAS which excels in identifying primarily non-functional common variants which may be correlated with causal variants. In this study, >95% of our analyzed variants were rare (MAF < 5%). Although we focused on variants more likely to be causal, it is possible that the sentinel SNPs identified in the GWAS were not tagging variants in the functional classes we examined, and that more genes would have reached significance if common, non-functional variation was included in our analysis. Further, the genes identified were in large cohorts of EA. Genetic differences between EA and South Asians, including allele frequency and linkage disequilibrium, could have contributed to the lack of findings. Another explanation is that genetic associations with cognitive function may be attenuated in this population due to more heterogeneity in environmental factors across India, such as sociodemographic factors, sociocultural factors, and air pollution, each of which is associated with cognitive function [72,73,74,75,76]. There is limited but expanding literature exploring the relationships between various sociocultural and environmental factors on cognitive function in India. While we adjust some sociodemographic factors such as literacy [77], rural/urban status [78], and educational attainment [79], as well as BMI and lifestyle factors, we do not model other emerging risk factors such as air pollution [73] and nutrition [75]. Additionally, new literature show that sociocultural factors in India may interact with genetic factors of cognitive function [80]. Finally, the tendency toward associations being nominally significant, but not significant after multiple testing corrections, may be a result of the smaller sample size.

One limitation of this study is that we examined only two classes of functional annotations. It may be that variants with other functional consequences besides missense/LoF and promoter/enhancer variants could influence associations with cognitive function. Another limitation is that we could not include insertion/deletion variants in this analysis, as annotation weights are not available; however, it is possible that these variants may have more deleterious effects on proteins and their removal may have attenuated signal. Additionally, the LASI-DAD cohort design oversamples LASI participants with higher cognitive impairment risk, which may result in different observed genetic associations with cognitive function, compared to studies sampled in other ways [48]. Finally, cognitive measures may have been biased due to administering the tests in many different languages [17]. However, no systematic bias with respect to language has been detected in LASI-DAD [81].

Our study also has several strengths. The prioritization and aggregation of SNVs based on their actual or predicted functional consequences likely increased signal for associations between the genes and cognitive function by focusing on variants that are more likely to have causal effects. Gene-based analysis with functional annotation also more directly links SNVs disease etiology, allowing a greater understanding of the types of variation within these genes that contribute to cognitive function. Additionally, to our knowledge, our study is the first to examine gene-based, rare variant associations with cognitive function in South Asians living in India. Thus, this work addresses an important health disparity in an understudied population [82]. Furthermore, our cohort presented a unique genetic environment to study potentially novel associations with understudied genetic variants due to its large genetic heterogeneity, unique subpopulations, and unique genetic ancestry [17,52,83]. Finally, we examined several measures of cognitive function, which allow us to determine which specific cognitive domains are associated with each gene.

In conclusion, we found that three genes (*APOE*, *PICALM*, and *TSPOAP1*) associated with Alzheimer’s disease in EA are also associated with the measure of cognitive functions in South Asians living in India, with the association primarily driven by missense/LoF SNVs. Associations were in part driven by rare, deleterious alleles, including a very rare SNV enriched in LASI-DAD compared to EA. Future functional studies are needed to verify and characterize SNVs found within this study.

## 5. Conclusions

Missense/LoF variants in some genes previously associated with AD in EA are associated with measures of cognitive function in South Asians from India. Analyzing genome sequence data allows identification of potential novel causal variants enriched in South Asians. Future functional studies are needed to verify and characterize SNVs found within this study.

## Figures and Tables

**Table 1:** Characteristics of the LASI-DAD analytic sample (*N* = 2680).

Covariate	Count (%) or Mean (SD)
Age (years)	69.6 (7.3)
Sex (female)	1408 (52.5)
Literacy (cannot read or write)	1511 (56.4)
Location	
Rural	1697 (63.3)
Urban	983 (36.7)
Education	
Less than lower secondary	2004 (75)
Upper secondary and vocational training	578 (22)
Tertiary	98 (4)
Body Mass Index (BMI) *	
Underweight (<18 kg/m^2^)	401 (16.2)
Normal weight (18 to <23 kg/m^2^)	1002 (40.5)
Overweight (23 to <25 kg/m^2^)	370 (15.0)
Obese (≥25 kg/m^2^)	701 (28.3)
Alcohol consumption *	
No	2482 (92.6)
Yes	183 (6.8)
Smoking *	
Never	2062 (76.9)
Former	175 (6.5)
Current	427 (15.9)
Physical activity *	
No	2231 (83.2)
Yes	433 (16.2)
Psychiatric medication use *	
No	2664 (99.4)
Yes	6 (0.2)
AD/dementia medication use *	
No	2661 (99.3)
Yes	8 (0.3)
HMSE score	22.7 (5.4)

HMSE = Hindi Mental State Exam, LASI-DAD = Longitudinal Aging Study in India—Diagnostic Assessment of Dementia, AD = Alzheimer’s Disease; * Percentages may not add to 100 due to missing data.

**Table 2:** Five-number summary of variants in missense/loss-of-function and promoter/enhancer analyses.

Analysis	Minimum	Q1	Median	Q3	Maximum	Number of Genes	Number of SNVs with MAF > 0	Total Number of SNVs Analyzed *
Missense/LoF	3	15	23	40	178	77	2510	2507
Missense	3	14	21	38	167	77	2442	2439
LoF	1	1	1	2	11	36	68	68
Promoter/Enhancer	6	61	93	127	265	77	7402	7370
Promoter	6	59	91	125	231	77	7108	7077
Enhancer	29	37.25	48	62	88	10	509	508

SNV = single nucleotide variant, MAF = minor allele frequency, LoF = loss-of-function, Q1 = quartile 1, Q3 = quartile 3. * Only SNVs with MAF > 0 and a complete set of annotation weights were analyzed. Total number of SNVs is the number of unique SNVs analyzed across all genes selected for analysis.

**Table 3:** Genes with FDR q < 0.1 in Missense/LoF Analysis.

Gene	Model	Number of SNVs Analyzed	*p*-Value (Without Annotation Weights)	*p*-Value (with Annotation Weights)
HMSE Score				
*APOE*	Model 1	20	9.5 × 10^−4^ *	0.001 *
*PICALM*	Model 1	16	0.002 *	0.002 *
*PICALM*	Model 2	16	0.001 *	0.001 *
General Cognitive Function				
*APOE*	Model 1	20	5.6 × 10^−4^ *	7.8 × 10^−4^ *
Executive Function				
*APOE*	Model 1	20	0.002 *	0.002
*TSPOAP1*	Model 1	89	0.002 *	0.004
Orientation				
*APOE*	Model 1	20	9.3 × 10^−4^ *	0.001 *

HMSE = Hindi Mental State Exam, FDR = false discovery rate. Model 1 adjusts for age, sex, state or union territory (as a fixed effect and with heterogeneous variances), the first ten genetic principal components, and genetic relatedness (matrix). Model 2 adjusts for all Model 1 covariates and educational attainment, rural or urban residence, and literacy status. Genes were included if either the *p*-value without annotation weights or *p*-value with annotation weights was <0.05 in Model 1. * FDR q-values < 0.1.

**Table 4:** Sentinel SNVs from significant genes in the missense/LoF analysis without annotation weights.

Cognitive Function	Model	rsID	ID	Gene	Allele (Effect Direction)	AF in LASI-DAD	AF in EA gnomAD	SNV Functional Annotation	Position in Gene	*p*-Value
HMSE Score	Model 1	rs429358	19:44908684:T:C	*APOE*	C (−)	0.11	0.15	Missense	Exon 4	2.9 × 10^−4^
HMSE Score	Model 1	rs779406084	11:85974781:G:A	*PICALM*	A (−)	0.00075	0.000015	Missense	Exon 19	4.2 × 10^−4^
HMSE Score	Model 2	rs779406084	11:85974781:G:A	*PICALM*	A (−)	0.00075	0.000015	Missense	Exon 19	1.6 × 10^−4^
General Cognitive Function	Model 1	rs429358	19:44908684:T:C	*APOE*	C (−)	0.11	0.15	Missense	Exon 4	1.4 × 10^−4^
Executive Function	Model 1	rs429358	19:44908684:T:C	*APOE*	C (−)	0.11	0.15	Missense	Exon 4	4.1 × 10^−4^
Executive Function	Model 1	rs9913145	17:58312371:T:C	*TSPOAP1*	C (+)	0.15	0.12	Missense	Exon 17	5.7 × 10^−4^
Orientation	Model 1	rs429358	19:44908684:T:C	*APOE*	C (−)	0.11	0.15	Missense	Exon 4	2.4 × 10^−4^

AF = allele frequency, HMSE = Hindi Mental State Exam, SNV = single nucleotide variant, FDR = false discovery rate, LoF = loss-of-function, EA = European ancestry, LASI-DAD = Longitudinal Aging Study in India—Diagnostic Assessment of Dementia. Model 1 adjusts for age, sex, state, or union territory (as a fixed effect and with heterogeneous variances), the first ten genetic principal components, and genetic relatedness (matrix). Model 2 adjusts for all Model 1 covariates and educational attainment, rural or urban residence, and literacy status.

## Data Availability

Whole genome sequencing data for the Diagnostic Assessment of Dementia for the Longitudinal Aging Study of India (LASI-DAD) is available from the National Institute on Aging Genetics of Alzheimer’s Disease Data Storage Site (NIAGADS), accession number: NG00067–ADSP Umbrella. Phenotype data are available at the Gateway to Global Aging website, https://g2aging.org/.

## Supplementary Materials

The following supporting information can be downloaded at: https://www.mdpi.com/article/10.3390/genes16060640/s1, Supplemental Methods: Gene Selection; Supplemental Methods: Annotation Scores; Table S1: Sources used to identify the 84 genes analyzed in this study; Table S2: Summary of cognitive measures in LASI-DAD; Table S3: Annotation weight distribution for missense/LoF SNVs; Table S4: Annotation weight distribution for promoter/enhancer SNVs; Table S5: Genes with at least one nominally significant association (*p* < 0.05) in missense/LoF analysis (Model 1); Table S6: Genes with at least one nominally significant association (*p* < 0.05) in missense/LoF analysis (Model 2); Table S7: Genes with at least one nominally significant (*p* < 0.05) association in the high-confidence missense/LoF SNV analysis (Model 1); Table S8: Genes with at least one nominally significant (*p* < 0.05) association in the high-confidence missense/LoF SNV analysis (Model 2); Table S9: Genes with at least one nominally significant (*p* < 0.05) association in the brain-specific promoter/enhancer analysis (Model 1); Table S10: Genes with at least one nominally significant (*p* < 0.05) association in the brain-specific promoter/enhancer analysis (Model 2); Figure S1: Genes nominally associated (p < 0.05) with at least one measure of cognitive function in the missense/LoF analysis without annotation weights; Figure S2: Genes nominally associated (p < 0.05) with at least one measure of cognitive function in the high-confidence missense/LoF analysis without annotation weights; Figure S3: Genes nominally associated (p < 0.05) with at least one measure of cognitive function in the promoter/enhancer analysis without annotation weights.

## Abbreviations

The following abbreviations are used in this manuscript:

AD	Alzheimer’s Disease
EA	European Ancestry
LASI-DAD	Harmonized Diagnostic Assessment of Dementia in the Longitudinal Aging Study of India
HMSE	Hindi Mental State Examination
LoF	Loss-of-Function
STAAR	variant-Set Test for Association using Annotation infoRmation
WGS	Whole Genome Sequencing
SNV	Single Nucleotide Variant
LASI	Longitudinal Aging Study of India
HCAP	Harmonized Cognitive Assessment Protocol
QC	Quality Control
GCAD	Genome Center for Alzheimer’s Disease
DP	Read Depth
GQ	Genotype Quality Score
VQSR	Variant Quality Score Recalibration
SNP	Single Nucleotide Polymorphism
PC	Principal Component
GRM	Genetic Relatedness Matrix
CHC	Cattell–Horn–Carroll
IRT	Item-Response Theory
GWAS	Genome-Wide Association Study
VEP	Variant Effect Predictor
ADD	AD and Dementia
WGSA	WGS Annotator
FDR	False-Discovery Rate
AF	Allele Frequency
MAF	Minor Allele Frequency
