# Supplementary material for: Region-Based Analysis with Functional Annotation Identifies Genes Associated with Cognitive Function in South Asians from India
Source: Genes (Basel). 2025 May 27;16(6):640. doi: 10.3390/genes16060640 (PMC12192162; doi:10.3390/genes16060640)
Supplement: Supplementary file 1 [file genes-16-00640-s001.zip › genes-3600033-supplementary.pdf]

Supplementary Methods

Methods S1: Gene Selection

We selected a total of 84 genes from EA GWAS of AD (Bellenguez et al.[1] and Wightman et al.[2]) as well as genes in the *APOE* region previously associated with AD (*TOMM40* and *APOC1*; Supplementary Table 1) [3–6] From Bellenguez et al. [1], we selected a total of 73 genes that were either identified as known loci (i.e., appeared in Bellenguez Table 1) or that had the strongest evidence of driving the detected novel AD associations (tier 1 in gene prioritization; see Bellenguez Supplementary Table 20). From Wightman et al. [2] we selected 45 genes from independent loci that reached genome-wide significance (see Wightman Table 1).

For any loci that were listed in the format "gene A/gene B", both genes were included in our analysis, and we examined the gene region to identify additional genes that may be relevant within the locus. Five loci from Bellenguez Table 1 and four loci from Wightman Table 1 were listed as "gene A/gene B", with an overlap of three loci between them. For the locus HLA from Bellenguez Table 1, we used *HLA-DQA1, HLA-DRB1*, and *HLA-DRB5* separately because this locus was labeled as *HLA-DRB1* and *HLA-DRB5* in other AD GWAS [7–10] and *HLA-DQA1* was labeled as the closest gene in Bellenguez et al.[1] For the locus MS4A, we used the genes *MS4A4A* and *MS4A6A* separately, as MS4A has been labeled as *MS4A6A* [8,9] *MS4A2* [7], and *MS4A4A* [1,2] in various AD GWAS. Finally, for *CELF1/SPI1*, we also used *CELF1, SPI1,* and *MADD* separately. This is due to the locus *CELF1/SPI1* being labeled as *CELF1* [9] *SPI1/CELF1* [7], and *MADD/SPI1[10]* in various AD GWAS. The *ZCWPW1/NYAP1* locus has been labeled as *ZCWPW1[8,9]* and *ZCWPW1/NYAP1[7,10]*. While *SPYDE3* was listed as the closest gene in Bellenguez [1] to the *ZCWPW1/NYAP1* locus, we did not examine this gene because AD GWAS

did not specify *SPYDE3* as a prioritized gene. The *SLC24A4/RIN3* locus has been labeled as *SLC24A4/RIN3* [9] *SLC24A4* [7,8] and *RIN3[10]* in previous GWAS.

Methods S2: Annotation Scores

For missense/LoF SNVs, we used CADD_raw_rankscore, a measure of variant deleteriousness combining multiple genomic features of each variant [11]; GERP_RS_rankscore, a measure of variant conservation [12]; Eigen.phred, a measure of variant deleteriousness using an unsupervised learning method [13]; and fathmm.MKL_coding_rankscore, a score from a machine learning method incorporating other annotations to predict deleteriousness of the variant from coding variants [14]. For promoter/enhancer SNVs, we used CADD_raw_rankscore [11], GERP_RS_rankscore [12], Eigen.PC.phred [13], fathmm.MKL_non.coding_rankscore [14], and GenoCanyon_rankscore, a measure of variant conservation [15]. The rank score variables are a number between zero and one indicating the genome-wide ranks of the corresponding annotation score across all variants as retrieved via WGS Annotator. Higher rank score variables indicate higher annotation scores. Per the STAAR tutorial, Phred scores were calculated from the rank scores according to the equation $-10*\log_{10}(1-x)$, where x is the rank score. For Eigen.phred and Eigen.PC.phred, these Phred scores were provided from the original database using either coding or non-coding variant training data. Almost all missense/LoF SNVs had Eigen.phred calculated using the coding training data set, representing a Phred score among all coding variants. Almost all promoter/enhancer SNVs had Eigen.PC.phred calculated with a non-coding variant training dataset, representing a Phred score among all non-coding variants.

Table S1: Sources used to identify the 84 genes analyzed in this study.

Gene	Locus	Location in Bellenguez et al. (2022)* [1]	Location in Wightman et al. (2021)* [2]
ABCA1	*ABCA1*	Tier 1	N/A
ABCA7	*ABCA7*	Table 1	Table 1
ABI3	*ABI3*	Table 1	Table 1
ACE	*ACE*	Table 1	Table 1
ADAM10	*ADAM10*	Table 1	Table 1
ADAM17	*ADAM17*	Tier 1	N/A
ADAMTS1	*ADAMTS1*	Table 1	N/A
AGRN	*AGRN*	N/A	Table 1
APH1B	*APH1B*	Table 1	Table 1
APOC1	*APOE*	N/A	N/A
APOE	*APOE*	N/A	Table 1
APP	*APP*	Tier 1	Table 1
BIN1	*BIN1*	Table 1	Table 1
BLNK	*BLNK*	Tier 1	N/A
CASS4	*CASS4*	Table 1	Table 1
CCDC6	*ANK3*	Tier 1	Table 1
CD2AP	*CD2AP*	Table 1	Table 1
CD33	*CD33*	N/A	Table 1
CELF1	*CELF1/SPI1*	Table 1	N/A
CLNK	*CLNK/HS3ST1*	Table 1	Table 1
CLU	*CLU*	Table 1	Table 1
CR1	*CR1*	Table 1	Table 1
CTSB	*CTSB*	Tier 1	N/A
CTSH	*CTSH*	Tier 1	N/A
DGKQ	*IDUA*	Tier 1	N/A
DOC2A	*DOC2A*	Tier 1	N/A
ECHDC3	*USP6NL/ECHDC3*	Table 1	Table 1
EGFR	*SEC61G*	Tier 1	N/A
EPHA1	*EPHA1/EPHA1-AS1*	Table 1	N/A
EPHA1-AS1	*EPHA1/EPHA1-AS1*	N/A	Table 1
FERMT2	*FERMT2*	Table 1	Table 1
GRN	*GRN*	Tier 1	Table 1
HAVCR2	*HAVCR2*	N/A	Table 1
HLA-DQA1	*HLA*	Table 1	Table 1
HLA-DRB1	*HLA*	Table 1	Table 1
HLA-DRB5	*HLA*	Table 1	Table 1
HS3ST1	*CLNK/HS3ST1*	Table 1	N/A
ICA1	*ICA1*	Tier 1	N/A
ICA1L	*WDR12*	Tier 1	N/A
IL34	*IL34*	Table 1	N/A
INPP5D	*INPP5D*	Table 1	Table 1
JAZF1	*JAZF1*	Tier 1	N/A
KAT8	*KAT8*	Table 1	N/A
LILRB2	*LILRB2*	Tier 2	Table 1

LIME1	*SLC2A4RG*	Tier 1	N/A
MADD	*CELF1/SPI1*	Table 1	Table 1
MAF	*MAF*	Tier 1	N/A
MAPT	*MAPT*	Table 1	N/A
MME	*MME*	Tier 1	N/A
MS4A4A	*MS4A*	Table 1	Table 1
MS4A6A	*MS4A*	Table 1	Table 1
MYO15A	*MYO15A*	Tier 1	N/A
NCK2	*NCK2*	Tier 1	Table 1
NME8	*NME8*	Table 1	N/A
NTN5	*NTN5*	N/A	Table 1
NYAP1	*ZCWPW1/NYAP1*	Table 1	Table 1
OTULIN	*ANKH*	Tier 1	N/A
PICALM	*PICALM*	Table 1	Table 1
PLCG2	*PLCG2*	Table 1	N/A
PLEKHA1	*PLEKHA1*	Tier 1	N/A
PTK2B	*PTK2B*	Table 1	N/A
RABEP1	*SCIMP/RABEP1*	Table 1	Table 1
RBCK1	*RBCK1*	Tier 1	N/A
RHOH	*RHOH*	Tier 1	N/A
RIN3	*SLC24A4/RIN3*	Table 1	Table 1
RITA1	*TPCN1*	Tier 1	N/A
SCIMP	*SCIMP/RABEP1*	Table 1	Table 1
SHARPIN	*SHARPIN*	Tier 1	Table 1
SIGLEC11	*SIGLEC11*	Tier 1	N/A
SLC24A4	*SLC24A4/RIN3*	Table 1	N/A
SORL1	*SORL1*	Table 1	Table 1
SORT1	*SORT1*	Tier 1	N/A
SPI1	*CELF1/SPI1*	Table 1	Table 1
SPPL2A	*SPPL2A*	Table 1	N/A
TMEM106B	*TMEM106B*	Tier 1	Table 1
TNIP1	*TNIP1*	Tier 1	Table 1
TOMM40	*APOE*	N/A	N/A
TREM2	*TREM2*	Table 1	Table 1
TSPAN14	*TSPAN14*	Tier 1	N/A
TSPOAP1	*TSPOAP1/TSPOAP1-AS1*	Table 1	N/A
TSPOAP1-AS1	*TSPOAP1/TSPOAP1-AS1*	N/A	Table 1
USP6NL	*USP6NL/ECHDC3*	N/A	Table 1
WDR81	*WDR81*	Tier 1	N/A
ZCWPW1	*ZCWPW1/NYAP1*	Table 1	Table 1

*Indicates the location or prioritization of each gene in the Bellenguez et al. (2022) [1] or Wightman et al. [2] (2021) genome-wide association study

Table S2: Summary of cognitive measures in LASI-DAD

Phenotype	Minimum	Q1	Median	Q3	Maximum	Mean	SD
HMSE score	0	19	24	27	30	22.7	5.39
General Cognitive Function	-3.03	-0.68	-0.044	0.68	2.77	0.010	0.92
Memory	-2.20	-0.63	-0.049	0.64	3.63	0.022	0.94
Executive Function	-1.93	-0.69	-0.067	0.65	2.48	-0.001	0.90
Orientation	-2.48	-0.55	-0.003	0.94	0.94	-0.020	0.79
Language/Fluency	-3.37	-0.55	0.003	0.55	1.96	-0.031	0.80
Visuospatial	-1.58	-0.73	-0.104	0.54	1.58	0.036	0.83

HMSE = Hindi Mental State Exam, SD = Standard Deviation, Q1 = Quartile 1, Q3 = Quartile 3

Table S3 Annotation weight distribution for missense/LoF SNVs.

Annotation	Minimum	Q1	Median	Q3	Maximum	Mean
CADD_raw_rankscore	0.00	0.95	0.99	1.00	1.00	0.87
fathmm_MKL_coding_rankscore	0.00	0.90	0.98	0.99	1.00	0.89
GERP_RS_rankscore	0.00	0.78	0.97	0.99	1.00	0.79
Eigen-Phred	0.00	0.70	2.21	4.74	27.05	3.24
Transformed Eigen-Phred*	0.00	0.15	0.40	0.66	1.00	0.42

*Transformed by applying the equation 1 – 10^(-(Eigen-Phred)/10) to compare to rank score variables on a scale from 0 to 1. Annotation weights were taken from unique variants in our analysis. Variants were analyzed if they had a minor allele frequency (MAF)>0 and a complete set of annotation weights.

Table S4: Annotation weight distribution for promoter/enhancer SNVs.

Annotation	Minimum	Q1	Median	Q3	Maximum	Mean
CADD_raw_rankscore	0.00	0.44	0.76	0.91	1.00	0.66
fathmm_MKL_non-coding_rankscore	0.00	0.57	0.79	0.91	1.00	0.72
GERP_RS_rankscore	0.00	0.14	0.45	0.85	1.00	0.49
GenoCanyon_score	0.32	0.93	0.97	0.97	1.00	0.94
Eigen-PC-Phred	0.00	10.53	16.80	22.51	48.24	16.60
Transformed Eigen-PC-Phred*	0.00	0.91	0.98	0.99	1.00	0.90

*Transformed by applying the equation 1 – 10^(-(Eigen-PC-Phred)/10) to compare to rank score variables on a scale from 0 to 1. Annotation weights were taken from unique variants in our analysis. Variants were analyzed if they had a minor allele frequency (MAF)>0 and a complete set of annotation weights.

Table S5: Nominally associated genes (p<0.05) in missense/LoF SNV analysis (Model 1)

Gene	Number of SNVs Analyzed	P-value (without annotation weights)	P-value (with annotation weights)
**HMSE Score**			
ADAM17	15	**0.017**	**0.021**
APOE	20	**$9.5x10^{-4}$***	**0.001***
PICALM	16	**0.002***	**0.002***
ABCA7	178	0.074	**0.039**
MS4A6A	16	0.063	**0.049**
**General Cognitive Function**			
OTULIN	15	**0.013**	**0.028**
APOE	20	**$5.6x10^{-4}$***	**$7.8x10^{-4}$***
LILRB2	69	**0.024**	0.066
TSPOAP1	89	**0.006**	**0.013**
ABCA7	178	0.051	**0.034**
**Memory**			
APOE	20	**0.002**	**0.002**
TSPOAP1	89	**0.004**	**0.007**
MAF	48	0.066	**0.048**
**Executive Function**			
ADAM17	15	**0.029**	**0.036**
APOE	20	**0.002***	**0.002**
MAPT	46	**0.045**	**0.047**
TSPOAP1	89	**0.002***	**0.004**
**Orientation**			
ABCA7	178	**0.018**	**0.008**
CCDC6	9	**0.022**	**0.022**
OTULIN	15	**0.025**	0.056
APOE	20	**$9.3x10^{-4}$***	**0.001**
MAF	48	**0.029**	**0.036**
TSPOAP1	89	**0.049**	0.106
ABI3	18	0.061	**0.031**
**Language/Fluency**			
OTULIN	15	**0.037**	0.082
APOE	20	**0.028**	**0.024**
CTSH	17	**0.019**	**0.029**
NTN5	36	**0.011**	**0.016**
SORL1	96	**0.022**	**0.031**
ECHDC3	21	**0.026**	**0.043**
**Visuospatial**			
ADAM17	15	**0.042**	0.052
CR1	77	**0.028**	**0.024**
NCK2	8	**0.009**	**0.010**

HMSE = Hindi Mental State Exam, FDR = False Discovery Rate
Model 1 adjusts for age, sex, state or union territory (as a fixed effect and with heterogeneous variances), the first ten genetic principal components, and genetic relatedness (matrix).
Genes were included if the P-value with or without annotation weights was <0.05 in Model 1.
P-values<0.05 are in bold.

*FDR q-values<0.1

Table S6: Nominally associated genes ($p<0.05$) in missense/LoF SNV analysis (Model 2)

Gene	Number of SNVs Analyzed	P-value (without annotation weights)	P-value (with annotation weights)
**HMSE Score**			
ABCA1	79	**0.017**§	**0.017**§
APOE	20	**0.010**§	**0.016**§
PICALM	16	**0.001***	**0.001***
MS4A6A	15	0.059	**0.045**
**General Cognitive Function**			
ABCA7	178	**0.016**§	**0.001**
CCDC6	9	**0.042**	**0.042**
APOE	20	**0.027**§	**0.039**§
MME	31	**0.026**§	**0.027**§
TSPOAP1	89	**0.017**	**0.024**
HLA-DRB5	16	0.070	**0.050**§
**Memory**			
CASS4	37	**0.030**§	**0.029**§
TSPOAP1	89	**0.014**	**0.022**
APOE	20	**0.030**§	**0.044**§
BLNK	12	0.053	**0.042**
**Executive Function**			
TSPOAP1	89	**0.011**	**0.015**
CCDC6	9	**0.020**	**0.021**
EPHA1-AS1	31	**0.033**§	**0.034**§
**Orientation**			
ABCA7	178	**0.009**	**0.005**
ABI3	18	**0.024**§	**0.013**
CCDC6	9	**0.008**	**0.008**
APOE	20	**0.006**§	**0.009**§
DGKQ	38	**0.029**	**0.033**
RBCK1	17	**0.034**§	**0.035**§
HLA-DRB5	16	0.064	**0.047**
**Language/Fluency**			
TSPOAP1	89	**0.050**	**0.031**
MME	31	**0.048**§	**0.049**§
TREM2	11	**0.040**	**0.039**
PLEKHA1	19	**0.035**	**0.036**
CTSH	17	**0.014**	**0.021**
SORL1	96	**0.006**§	**0.006**§
**Visuospatial**			
ABCA1	79	**0.042**§	**0.031**
ADAM10	14	**0.025**§	1
ADAM17	15	**0.046**	0.057
APOE	20	**0.028**	**0.022**
NCK2	8	**0.034**	**0.038**
PLEKHA1	19	**0.014**	**0.015**
NTN5	36	0.067	**0.034**

HMSE = Hindi Mental State Exam, FDR = False Discovery Rate

Model 2 adjusts for all Model 1 covariates and educational attainment, rural or urban residence, and literacy status.
Genes were included if the P-value with or without annotation weights was <0.05 in Model 2.
P-values<0.05 are in bold.
*FDR q-values<0.1.
§No longer nominally significant ($p \geq 0.05$) in Model 3, which adjusts for all Model 2 covariates and BMI, physical activity, alcohol use, smoking, psychiatric medication use, and Alzheimer's Disease/dementia medication use.

Table S7: Nominally associated genes ($p<0.05$) in high-confidence missense/LoF SNV analysis (Model 1)

Gene	Number of SNVs Analyzed	P-Value (No Annotation Weights)	P-Value (Annotation Weights)
**General Cognitive Function**			
INPP5D	8	**0.028**	**0.026**
**Executive Function**			
ADAMTS1	13	**0.028**	**0.030**
INPP5D	8	**0.038**	**0.035**
**Orientation**			
ABCA7	63	**0.016**	**0.021**
ABI3	3	**0.044**	**0.045**
**Language/Fluency**			
APOE	7	**0.018**	**0.018**
**Visuospatial**			
ICA1	11	**0.026**	**0.024**
INPP5D	8	**0.022**	**0.020**

HMSE = Hindi Mental State Exam

Model 1 adjusts for age, sex, state or union territory (as a fixed effect and with heterogeneous variances), the first ten genetic principal components, and genetic relatedness (matrix).

Genes were included if the P-value with or without annotation weights was less than 0.05 Bolded values are $p<0.05$.

Table S8: Nominally associated genes ($p<0.05$) in high-confidence missense/LoF SNV analysis (Model 2)

Gene	Number of SNVs Analyzed	P-Value (No Annotation Weights)	P-Value (Annotation Weights)
**Executive Function**			
INPP5D	8	0.051	**0.048**§
**Orientation**			
ABI3	3	**0.039**§	**0.040**§
SLC24A4	11	**0.048**§	**0.043**§
**Visuospatial**			
ABCA1	36	**0.035**§	**0.039**§
APOE	7	**0.015**	**0.015**
GRN	10	**0.025**§	**0.025**§
INPP5D	8	**0.041**	**0.039**

HMSE = Hindi Mental State Exam
Model 2 adjusts for all Model 1 covariates and educational attainment, rural or urban residence, and literacy status.
Genes were included if the P-value with or without annotation weights was less than 0.05 Bolded values are $p<0.05$.
§No longer nominally significant ($p\geq0.05$) in Model 3, which adjusts for all Model 2 covariates and BMI, physical activity, alcohol use, smoking, psychiatric medication use, and Alzheimer's Disease/dementia medication use, or no longer analyzable under Model 3 due to number of variants<2 or cumulative minor allele count<5.

Table S9: Nominally associated genes in the Brain-Specific Promoter/Enhancer analysis in Model 1.

Gene	Number of SNVs Analyzed	P-value (without annotation weights)	P-value (with annotation weights)
**HMSE Score**			
APOE	101	**0.017**	**0.018**
CCDC6	125	**0.017**	**0.021**
PLCG2	146	**0.046**	**0.045**
SCIMP	28	**0.037**	**0.019**
**General Cognitive Function**			
APOE	101	**0.008**	**0.009**
BLNK	98	**0.044**	**0.038**
KAT8	52	**0.040**	0.055
SCIMP	28	**0.022**	**0.026**
TSPOAP1	231	**0.020**	**0.021**
TSPOAP1-AS1	158	**0.009**	**0.011**
APOC1	93	0.060	**0.050**
**Memory**			
APOC1	93	**0.037**	**0.029**
APOE	101	**0.026**	**0.028**
INPP5D	168	**0.047**	0.066
JAZF1	71	**0.025**	**0.027**
SCIMP	28	**0.006**	**0.008**
TSPOAP1	231	**0.006**	**0.006**
TSPOAP1-AS1	158	**0.003**	**0.004**
**Executive Function**			
APOE	101	**0.025**	**0.027**
BIN1	213	**0.013**	**0.012**
CD33	23	**0.040**	**0.016**
MAPT	151	**0.026**	**0.026**
SHARPIN	116	**0.033**	**0.031**
TMEM106B	129	**0.020**	**0.020**
TSPOAP1	231	**0.019**	**0.022**
TSPOAP1-AS1	158	**0.012**	**0.015**
**Orientation**			
ADAM17	80	**0.027**	**0.022**
APOE	101	**0.015**	**0.017**
SCIMP	28	**0.023**	**0.026**
USP6NL	100	**0.037**	0.083
**Language/Fluency**			
CTSH	65	**0.012**	**0.011**
HLA-DQA1	26	**0.049**	**0.043**
HLA-DRB1	44	**0.022**	**0.024**
PLCG2	146	**0.009**	**0.009**
SCIMP	28	**0.016**	**0.017**
**Visuospatial**			
BLNK	98	**0.046**	**0.028**

HMSE = Hindi Mental State Exam, FDR = False Discovery Rate
Model 1 adjusts for age, sex, state or union territory (as a fixed effect and with heterogeneous variances), the first ten genetic principal components, and genetic relatedness (matrix).
Genes were included if the P-value with or without annotation weights was <0.05 in Model 1.
P-values<0.05 are in bold.
*FDR q-values<0.1.

Table S10: Nominally associated genes in Brain-Specific Promoter/Enhancer analysis in Model 2.

Gene	Number of SNVs	P-value (without annotation weights)	P-value (with annotation weights)
**HMSE Score**			
BLNK	98	**0.039**§	**0.036**§
ADAM17	80	0.052	**0.044**§
**General Cognitive Function**			
ADAM10	133	**0.040**§	**0.038**§
ADAM17	80	**0.026**	**0.020**
BLNK	98	**0.007**	**0.006**
FERMT2	132	**0.036**§	**0.045**§
TMEM106B	129	**0.023**	**0.023**
**Memory**			
ADAM10	133	**0.049**§	0.095
JAZF1	71	**0.012**	**0.013**
SCIMP	28	**0.008**	**0.012**
TSPOAP1	231	**0.019**	**0.021**
TSPOAP1-AS1	158	**0.008**	**0.011**
APOC1	93	0.053	**0.047**§
**Executive Function**			
ADAM17	80	**0.036**	**0.028**
BLNK	98	**0.029**	**0.024**
CD2AP	81	**0.034**§	**0.032**§
MAPT	151	**0.042**	**0.043**
RHOH	6	**0.010**	**0.014**
TMEM106B	129	**0.042**	0.051
TSPOAP1-AS1	158	**0.046**	0.055
**Orientation**			
ADAM17	80	**0.010**	**0.007**
BLNK	98	**0.049**§	**0.045**§
EGFR	127	**0.049**§	0.064
FERMT2	132	**0.018**§	**0.023**§
MAF	225	**0.024**§	**0.033**§
TOMM40	100	**0.044**§	**0.035**§
APOC1	93	0.060	**0.033**§
**Language/Fluency**			
CTSH	65	**0.006**	**0.006**
FERMT2	132	**0.004**§	**0.005**§
HLA-DQA1	26	**0.031**	**0.023**
INPP5D	168	**0.029**§	**0.043**§
PLCG2	146	**0.012**	**0.012**
SORT1	200	0.96	**0.036**§
**Visuospatial**			
APOE	101	**0.048**§	0.053
CD2AP	81	**0.015**	**0.014**
CR1	36	**0.030**§	**0.042**§

MS4A6A	59	**0.048**	**0.048**
TMEM106B	129	**0.032**	**0.028**

HMSE = Hindi Mental State Exam, FDR = False Discovery Rate
Model 2 adjusts for all Model 1 covariates and educational attainment, rural or urban residence, and literacy status.
Genes were included if the P-value with or without annotation weights was <0.05 in Model 2.
P-values<0.05 are in bold.
*FDR q-values<0.1.
§No longer nominally significant (p≥0.05) in Model 3, which adjusts for all Model 2 covariates and BMI, physical activity, alcohol use, smoking, psychiatric medication use, and Alzheimer's Disease/dementia medication use.

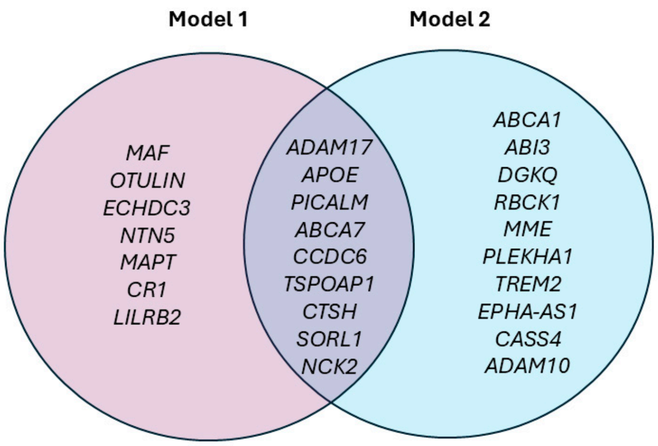

Figure S1: Genes nominally associated (p<0.05) with at least one measure of cognitive function in the missense/LoF analysis without annotation weights.

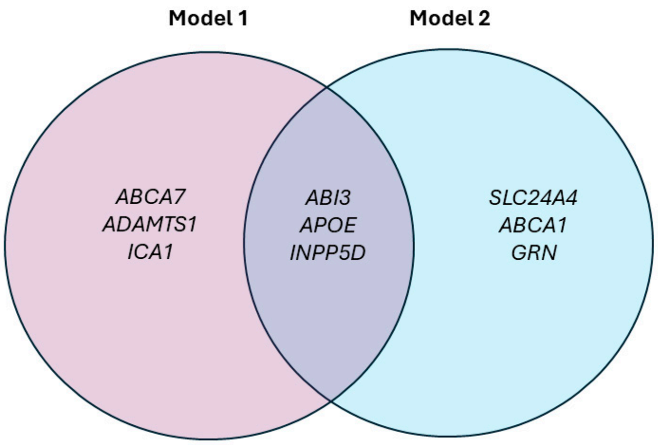

Figure S2: Genes nominally associated (p<0.05) with at least one measure of cognitive function in the high-confidence missense/LoF analysis without annotation weights.

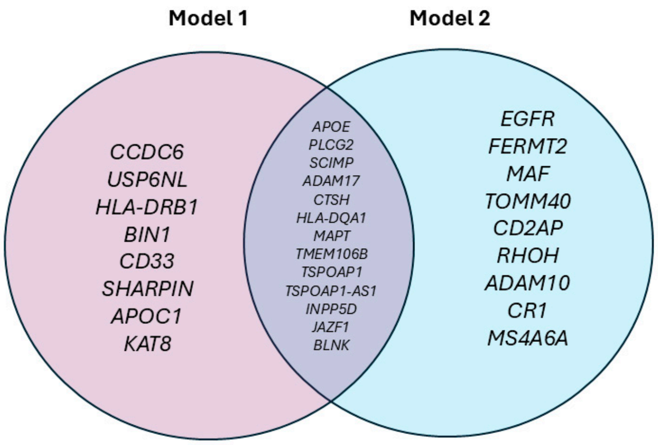

Figure S3: Genes nominally associated (p<0.05) with at least one measure of cognitive function in the promoter/enhancer analysis without annotation weights.

References

1. Bellenguez, C.; Küçükali, F.; Jansen, I.E.; Kleineidam, L.; Moreno-Grau, S.; Amin, N.; Naj, A.C.; Campos-Martin, R.; Grenier-Boley, B.; Andrade, V.; et al. New Insights into the Genetic Etiology of Alzheimer's Disease and Related Dementias. *Nat Genet* **2022**, *54*, 412–436, doi:10.1038/s41588-022-01024-z.

2. Wightman, D.P.; Jansen, I.E.; Savage, J.E.; Shadrin, A.A.; Bahrami, S.; Holland, D.; Rongve, A.; Børte, S.; Winsvold, B.S.; Drange, O.K.; et al. A Genome-Wide Association Study with 1,126,563 Individuals Identifies New Risk Loci for Alzheimer's Disease. *Nat Genet* **2021**, *53*, 1276–1282, doi:10.1038/s41588-021-00921-z.

3. Zhou, Q.; Zhao, F.; Lv, Z.P.; Zheng, C.G.; Zheng, W.D.; Sun, L.; Wang, N.N.; Pang, S.; De Andrade, F.M.; Fu, M.; et al. Association between APOC1 Polymorphism and Alzheimer's Disease: A Case-Control Study and Meta-Analysis. *PLoS One* **2014**, *9*, doi:10.1371/journal.pone.0087017.

4. Corder, E.H.; Saunders, A.M.; Strittmatter, W.J.; Schmechel, D.E.; Gaskell, P.C.; Small, G.W.; Roses, A.D.; Haines, J.L.; Pericak-Vance, M.A. Gene Dose of Apolipoprotein E Type 4 Allele and the Risk of Alzheimer's Disease in Late Onset Families. *Science (1979)* **1993**, *261*, 921–923, doi:10.1126/science.8346443.

5. Lutz, M.W.; Crenshaw, D.; Welsh-Bohmer, K.A.; Burns, D.K.; Roses, A.D. New Genetic Approaches to AD: Lessons from APOE-TOMM40 Phylogenetics. *Curr Neurol Neurosci Rep* **2016**, *16*, 48, doi:10.1007/s11910-016-0643-8.

6. Lin, R.; Zhang, Y.; Yan, D.; Liao, X.; Gong, G.; Hu, J.; Fu, Y.; Cai, W. Association of Common Variants in TOMM40/APOE/APOC1 Region with Human Longevity in a Chinese Population. *J Hum Genet* **2016**, *61*, 323–328, doi:10.1038/jhg.2015.150.

7. Kunkle, B.W.; Grenier-Boley, B.; Sims, R.; Bis, J.C.; Damotte, V.; Naj, A.C.; Boland, A.; Vronskaya, M.; van der Lee, S.J.; Amlie-Wolf, A.; et al. Genetic Meta-Analysis of Diagnosed Alzheimer's Disease Identifies New Risk Loci and Implicates Aβ, Tau, Immunity and Lipid Processing. *Nat Genet* **2019**, *51*, 414–430, doi:10.1038/s41588-019-0358-2.

8. Jansen, I.E.; Savage, J.E.; Watanabe, K.; Bryois, J.; Williams, D.M.; Steinberg, S.; Sealock, J.; Karlsson, I.K.; Hägg, S.; Athanasiu, L.; et al. Genome-Wide Meta-Analysis Identifies New Loci and Functional Pathways Influencing Alzheimer's Disease Risk. *Nat Genet* **2019**, *51*, 404–413, doi:10.1038/s41588-018-0311-9.

9. Lambert, J.C.; Ibrahim-Verbaas, C.A.; Harold, D.; Naj, A.C.; Sims, R.; Bellenguez, C.; DeStafano, A.L.; Bis, J.C.; Beecham, G.W.; Grenier-Boley, B.; et al. Meta-Analysis of 74,046 Individuals Identifies 11 New Susceptibility Loci for Alzheimer's Disease. *Nat Genet* **2013**, *45*, 1452–1458, doi:10.1038/ng.2802.

10. Wightman, D.P.; Jansen, I.E.; Savage, J.E.; Shadrin, A.A.; Bahrami, S.; Holland, D.; Rongve, A.; Børte, S.; Winsvold, B.S.; Drange, O.K.; et al. A Genome-Wide Association Study with 1,126,563 Individuals Identifies New Risk Loci for Alzheimer's Disease. *Nat Genet* **2021**, *53*, 1276–1282, doi:10.1038/s41588-021-00921-z.

11. Rentzsch, P.; Witten, D.; Cooper, G.M.; Shendure, J.; Kircher, M. CADD: Predicting the Deleteriousness of Variants throughout the Human Genome. *Nucleic Acids Res* **2019**, *47*, D886–D894, doi:10.1093/nar/gky1016.

12. Davydov, E. V.; Goode, D.L.; Sirota, M.; Cooper, G.M.; Sidow, A.; Batzoglou, S. Identifying a High Fraction of the Human Genome to Be under Selective Constraint Using GERP++. *PLoS Comput Biol* **2010**, *6*, doi:10.1371/journal.pcbi.1001025.

13. Ionita-Laza, I.; Mccallum, K.; Buxbaum, J. A SPECTRAL APPROACH INTEGRATING FUNCTIONAL GENOMIC ANNOTATIONS FOR CODING AND NONCODING VARIANTS IULIANA IONITA-LAZA HHS Public Access Author Manuscript. *Nat Genet* **2016**, *48*, 214–220, doi:10.1038/ng.3477.A.

14. Shihab, H.A.; Rogers, M.F.; Gough, J.; Mort, M.; Cooper, D.N.; Day, I.N.M.; Gaunt, T.R.; Campbell, C. An Integrative Approach to Predicting the Functional Effects of Non-Coding and Coding Sequence Variation. *Bioinformatics* **2015**, *31*, 1536–1543, doi:10.1093/bioinformatics/btv009.

15. Lu, Q.; Hu, Y.; Sun, J.; Cheng, Y.; Cheung, K.H.; Zhao, H. A Statistical Framework to Predict Functional Non-Coding Regions in the Human Genome through Integrated Analysis of Annotation Data. *Sci Rep* **2015**, *5*, 1–13, doi:10.1038/srep10576.
